# Circulating microRNA Associated to Different Stages of Liver Steatosis in Prader–Willi Syndrome and Non-Syndromic Obesity

**DOI:** 10.3390/jcm9041123

**Published:** 2020-04-14

**Authors:** Muhammad Yogi Pratama, Devis Pascut, Sofia Tamini, Alessandro Minocci, Claudio Tiribelli, Graziano Grugni, Alessandro Sartorio

**Affiliations:** 1 Fondazione Italiana Fegato – ONLUS, 34149 Trieste, Italy; yogi.pratama@fegato.it (M.Y.P.); ctliver@fegato.it (C.T.); 2 Faculty of Medicine, Universitas Hasanuddin, Makassar 90245, Indonesia; 3 Experimental Laboratory for Auxo-endocrinological Research, Istituto Auxologico Italiano, IRCCS, 28824 Piancavallo (VB), Italy; s.tamini@auxologico.it (S.T.); a.minocci@auxologico.it (A.M.); g.grugni@auxologico.it (G.G.); sartorio@auxologico.it (A.S.); 4 Division of Metabolic Diseases, Istituto Auxologico Italiano, IRCCS, 28824 Piancavallo (VB), Italy; 5 Division of Auxology, Istituto Auxologico Italiano, IRCCS, 28824 Piancavallo (VB), Italy

**Keywords:** microRNA, miRNA, Prader–Willi syndrome, steatosis, obese, non-syndromic obesity, serum mirna

## Abstract

Background: Prader–Willi syndrome (PWS) is a rare and poorly characterized disease. Recent genomic and transcriptomic studies contributed to elucidate the molecular bases of the syndrome. In this study, we characterized the expression of circulating miRNAs in patients with PWS compared to those with non-syndromic obesity in association with liver steatosis. Methods: MiRNAs were studied by qRT-PCR in serum samples from 30 PWS and 30 non-syndromic obese subjects. Results: MiRNA expression was associated with the presence of the syndrome and to the grade of liver steatosis. MiR-122-5p, miR-151a, miR-92a-3p were up-regulated in obese (4.38-fold, *p* < 0.01; 2.72-fold, *p* < 0.05; 1.34-fold *p* < 0.05, respectively) and were able to differentiate obese from PWS (AUC = 0.81, sens/spec 78/71%). When stratifying groups according to the presence of steatosis, the expression of miR-151a-5p, miR-92a-3p, miR-106b-5p, and miR-93-5p were lower in PWS with steatosis grade 1. Within the group with steatosis grade 1, miR-151a-5p was significantly distinguished PWS from obese (AUC = 0.85, sens/spec 80/85%) and the combination of miR-106b-5p and miR-93-5p showed higher performances in discriminating different grades of steatosis in PWS (AUC = 0.84, sens/spec 93/74%). Conclusions: MiRNAs represent a tool to better classify and characterize PWS, providing new information about the clinical picture and the extent of steatosis.

## 1. Introduction

Prader–Willi syndrome (PWS) is a complex genetic disorder due to the lack of expression of genes located on the paternal chromosome 15q11.2-q13. Approximately 65–70% of the affected subjects have a deletion of this area (del15), while 20–30% of patients present a maternal uniparental disomy involving the same region (UPD15), and few cases (2–5%) result from an imprinting center defect or other chromosomal rearrangement in chromosome 15 [1].

PWS represents the single most common known genetic cause of obesity with an estimated population prevalence varying from 1:10,000–1:30,000 live births [2]. The clinical characteristics of PWS includes neonatal hypotonia with initial feeding problems, early development of severe hyperphagia with food-seeking behavior, multiple hormonal abnormalities, dysmorphic features, autonomic dysregulation, mental retardation, and behavioral and psychiatric problems [3], accounting for a complex hypothalamic dysfunction [4]. Subjects with PWS have an elevated risk of developing severe obesity unless their food intake is strictly controlled. Obesity of PWS subjects shows distinct phenotypic and metabolic characteristics that are not common to non-syndromic obesity [5]. In fact, PWS harbors a higher fat mass than simple obesity at the same degree of weight excess [6]. Moreover, lean body mass is lower in PWS subjects than the body mass index (BMI)-matched population [7]. Excessive fat mass in PWS typically affects the trunk and the proximal extremity of the limbs, with lower trunk-to-appendicular fat mass ratio and lower visceral adiposity in comparison to non-syndromic obesity [8,9]. In line with these findings, insulin levels and insulin resistance are usually lower in PWS patients than in obese subjects [8,10]. Accordingly, the overall risk of glucose abnormalities is less marked than in controls with comparable BMI, but worsens with aging and weight accumulation [11]. Similarly, the risk of metabolic syndrome in obese PWS adults is very close to what reported in obese controls, suggesting the crucial role of obesity status [12]. By contrast, severe non-alcoholic fatty liver disease is less frequent in PWS patients, both in children and adults [13,14].

In addition to these major metabolic differences, some genomic and transcriptomic studies contributed to the molecular characterization of the disease [15,16,17]. In our previous work, we identified differences in the circulatory miRNome of PWS compared to non-syndromic obesity that might reflect the different regulatory pathways involved in the two diseases [17]. The relevance of circulating miRNAs as disease biomarkers have been extensively investigated in many clinical settings, suggesting their potential use as molecular tools for the identification of the molecular alterations characterizing the different pathological conditions.

Based on the above considerations, aim of this study is to characterize the expression of circulating miRNAs in patients with PWS compared to those with non-syndromic obesity, analyzing their association with the different stages of liver steatosis.

## 2. Materials and Methods

### 2.1. Patients

An experienced radiologist performed liver ultrasonography using standardized criteria [18,19] in 46 consecutive patients with PWS (16M/30F), hospitalized for a 3-week multidisciplinary body weight reduction program at the Division of Auxology, and in 42 consecutive patients (18M/24F) with non-syndromic obesity (OB), hospitalized for a multidisciplinary 3-week body weight reduction program at the Division of Metabolic Diseases, Istituto Auxologico Italiano, IRCCS, Piancavallo (VB), Italy. Thirty Caucasian PWS patients were found to have different degrees of liver steatosis (10 males, 20 females, age range: 18.7–56.7 yrs, mean ± s.d.: 35.0 ± 9.9 yrs; BMI range: 19.2–59.1 kg/m^2^, mean BMI: 39.8 ± 10.7 kg/m^2^) and were included in the study (Supplementary Table S1). All subjects showed the typical PWS clinical phenotype [20]. Among the selected patients, 21 (14 females) had del15, 7 (6 females) UPD15, while a positive methylation test was demonstrated in the remaining 2 (males) PWS cases, but the underlying genetic defect was not identified. Fifteen of 20 PWS women had primary amenorrhea, 9 females and 2 males were undergoing sex steroid replacement treatment. At the time of the study, 9 individuals were on growth hormone treatment, while 6 had been treated in the past. Fifteen PWS had never received growth hormone therapy. Nine PWS subjects suffered from hypothyroidism (8 females) and were biochemically euthyroid on thyroxine substitution. Eleven patients (9 females) were under therapy for type 2 diabetes mellitus (oral hypoglycemic agents and/or insulin). None of the PWS subjects suffered from central adrenal insufficiency. Thirty age-matched OB with liver steatosis (15 males, 15 females, age range: 20.5-55.6–yrs, mean ± s.d.: 37.7 ± 7.9 yrs; BMI range: 35.6–50.0 kg/m^2^, mean BMI: 41.0 ± 3.3 kg/m^2^) were also included in the study (Supplementary Table S1). All females had regular menses (3 were on estrogen treatment for contraception). Apart from severe obesity, no overt endocrine disorders (diabetes mellitus, hypothyroidism, hypercortisolism, etc.,) were present.

Physical examination included determination of height, weight, waist, and hip circumference in fasting conditions and after voiding. Standing height was determined by a wall-mounted Harpenden Stadiometer (Holtain Limited, Crymych, Dyfed, UK). Body weight was measured with subjects in minimal clothes to the nearest 0.1 kg, using standard equipment. A BMI cut-off point of 30 kg/m^2^ was used to define obesity. Waist and hip circumferences were measured with a flexible tape measure with the subject standing erect and relaxed with arms at the sides and feet positioned closed together. Waist circumference was measured at the midpoint between the iliac crest and last rib, hip circumference was measured at the widest part of the hip at the level of the greater trochanter.

The evaluation of fat free mass (FFM) and fat mass (FM) was performed throughout bio-impedentiometry (Human-IM Touch, DS-Medigroup, Milan, Italy). Diastolic and systolic blood pressure were measured, in the supine position, twice (3-min interval in-between) on the dominant arm with an aneroid sphygmomanometer (TemaCertus, Milan, Italy), by using appropriate sized cuffs. The mean values were calculated and rounded to the nearest 5 mmHg value.

The study was approved by the Ethics Committee of Istituto Auxologico Italiano, IRCCS, Milan, Italy (ref. no. 01C823; acronym: MIRNOMAPWS). Before the study began, the purpose and objectives had been carefully explained to each patient. Written informed consent was obtained from all participants and their parents (when appropriate).

### 2.2. Serum Collection

Fasting blood samples were collected by standard venipuncture in BD Vacutainer^®^ serum separating tubes (BD - Plymouth PL6 7BP, UK) and centrifuged at 1900 × *g* at 4 °C for 10 min and at 16,000 × *g* at 4 °C for further 10 min. Supernatants were transferred into new tubes and subsequently frozen at –80 °C for long-term storage.

### 2.3. Assessment of Hemolysis

Hemoglobin was assessed with Beckman Coulter^®^ DU^®^730 spectrophotometer (Beckman Coulter, Brea, CA, USA) using the Harboe Direct spectrophotometric method [21] with Allen correction [22]: Hb (g/L) = (167.2 × A_415_ – 83.6 × A_380_ – 83.6 × A_450_) x 1/1000 × 1/dilution in dH_2_O. The considered cut-off for serum was 0.020g/L.

### 2.4. RNA Extraction

Small RNAs were isolated from 300 µL of serum using the Nucleospin™ miRNA Plasma Isolation Kits – Biofluids (Macherey-Nagel, Germany). MicroRNAs were quantified in a Qubit^®^ 2.0 Fluorometer (Thermo Fischer Scientific, Waltham, MA, USA) using the Qubit microRNA Assay Kit (Thermo Fischer Scientific, Waltham, MA, USA) following the manufacturer instructions.

### 2.5. qRT-PCR

Thirty nanograms of microRNAs were reverse transcribed by using the qScript microRNA cDNA Synthesis Kit (Quantbio, Beverly, MA, USA) according to manufacturer’s instruction. Samples were analyzed thought RT-qPCR by using the PerfeCTa SYBR^®^ Green SuperMix (Quantbio, Beverly, MA, USA) in a CFX-96 thermal cycler (Bio-Rad Laboratories, Hercules, CA, USA) according to the manufacturer’s instructions. All reactions were run in duplicate in a 25-uL reaction. MiRNA primers were purchased from Sigma-Aldrich (Merck KGaA, Darmstadt, Germany). MiR-1275 was used as endogenous normalizer. Expression levels were calculated by using the 2-ΔΔCt formula.

### 2.6. Data Analysis and Statistical Methods

The Mann-Whitney U test was used to compare the differences between the two independent groups. For multiple comparisons, Kruskal-Wallis test in One-Way ANOVA procedure was used. The receiver operating characteristic (ROC) curves were plotted to estimate the discriminatory potential of the miRNAs. A hierarchical forward selection with switching one-way logistic analysis was used to estimate the discriminatory potential of the miRNA combination. Analyses were performed using NCSS 11 Software (2016) (NCSS, LLC. Kaysville, UT, USA, ncss.com/software/ncss) and Stata 16.0 (Stata Corporation, College Station, TX, USA).

## 3. Results

### 3.1. Patient Characteristics

The clinical characteristics of PWS and OB are reported in Supplementary Table S1. The two subgroups were comparable for age, BMI, waist, and hip circumferences, body composition, systolic and diastolic blood pressure, while significant differences were found for height and weight, both lower in PWS. A better lipid profile was found in PWS, characterized by lower triglycerides levels and higher HDL cholesterol values, than that recorded in patients with OB (Supplementary Table S2). Insulin and homocysteine were significantly lower in PWS, whereas HbA1c and vitamin D were higher than in obese individuals. Liver enzymes were lower in PWS subjects compared to the control group. Degree 1 of liver steatosis was found in 33% of patients with OB (vs. 50% with PWS), degree 2 in 27% (vs. 37%), and degree 3 in 40% (vs. 13%), showing a lower liver impairment in our PWS population (Supplementary Table S1).

### 3.2. Selection of Differently Expressed miRNAs

The aim of this study is to clarify the role of previously identified circulating miRNAs [17] distinguishing PWS from OB subjects. Eight miRNA candidates differently expressed between the two groups with a fold of change (FC) greater than 6 were selected: MiR-23a-3p, mir-122-5p miR-24-3p, miR-93-5p, miR-92a-3p, miR-106b-5p, miR-425-5p, and miR-191-5p. Besides, miR-151a-5p was included in the miRNA candidate panel because of its exclusive expression in OB.

### 3.3. Differently Expressed miRNAs between PWS and OB Subjects

In the validation phase, the expression of the miRNA candidates was assessed in 30 age-matched samples of PWS and OB subjects by using qRT-PCR. Among the miRNAs tested, the expression of miR-122-5p, miR-151a, and miR-92a-3p were significantly up-regulated in OB subjects showing 4.38-fold (*p* < 0.01), 2.72-fold (*p* < 0.05), and 1.34-fold (*p* < 0.05) increase, respectively (Table 1). There were no significant differences for the remaining seven miRNAs (miR-425-5p, miR-23a-3p, miR-24-3p, miR-106b-5p, miR-191-5p, and miR-93-5p), although a nearly significant downregulation was observed for miR-425-5p (*p* = 0.06) in PWS.

**Figure 1:**
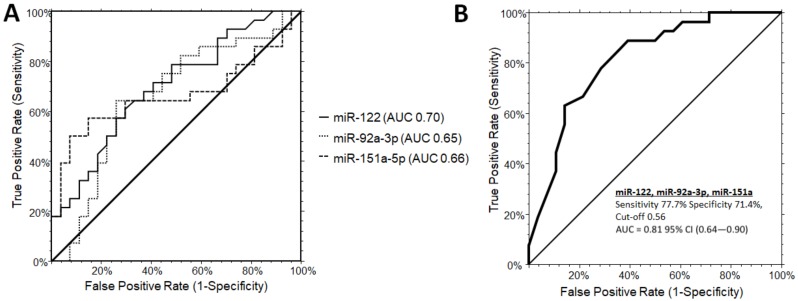
Receiver operating characteristic (ROC) curve analysis for single microRNAs (**A**) and combinations (B: miR-122-5p, miR-151a, miR-92a-3p) to discriminate PWS from OB subjects. (**B**) The AUC for the combination of the three miRNA was determined as 0.81 (0.64-0.90, 95% CI) with a sensitivity of 77.7% and a specificity of 71.4%, at a cut-off 0.56 (logit model formula: –1.34 + 2.97 × miR-122-5p - 0.57 × miR-92a_3p + 1.62 × miR-151a-5p). AUC, area under the ROC curve; CI, confidence interval.

The three significant miRNAs were included in a one-way stepwise logistic regression analysis to evaluate the discriminatory potential of miR-122-5p, miR-151a-5p, and miR-92a-3p in distinguishing PWS from OB subjects. Independent ROC curve analysis for the single miRNA candidate did not reach satisfactory performances. MiR-122-5p was the only candidate reaching the area under the curve (AUC) of the receiver operator characteristic (ROC) of 0.70 (0.52–0.81, 95% CI), while miR-92a-3p and miR-151a displayed an inferior AUC values of 0.65 (0.47–0.78, 95% CI) and 0.66 (0.48–0.79, 95% CI), respectively (Figure 1A). By combining miR-122-5p, miR-151a-5p, and miR-92a-3p, the AUC increased up to 0.81 (0.64–0.90, 95% CI) with a sensitivity of 77.7% and a specificity of 71.4%, at a cut-off determined at 0.56 (logit model formula: –1.34 + 2.97 × miR-122-5p - 0.57 × miR-92a_3p + 1.62 × miR-151a-5p) (Figure 1B).

### 3.4. Differently Expressed miRNAs between PWS and OB Subjects According to the Degree of Steatosis

Considering the interest in exploring the differences in the circulating miRNAs in relation to the grade of steatosis, the expression profiles between PWS and OB with steatosis (respectively PWS+ and OBS+) grade 1 were compared. MiR-151a, mir-92a-3p, miR-106b-5p, and miR-93-5p were identified as significantly upregulated in OBS+ grade 1 with a mean fold of change of 2.92-fold (*p* < 0.005), 1.91-fold (*p* < 0.01), 1.76-fold (*p* < 0.05), and 1.59-fold (*p* < 0.05), respectively (Table 2, Figure S1A–D).

**Figure 2:**
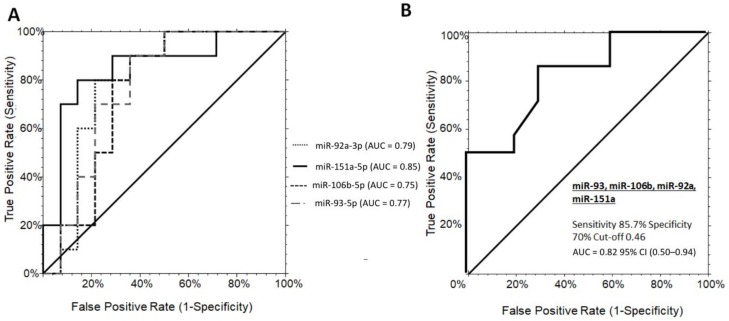
ROC curve analysis of serum miRNAs discriminating grade 1 PWS+ from OBS+. (**A**) ROC curves for the single miRNA candidates, miR-151a, miR-92a-3p, miR-106b-5p, and miR-93-5p with optimal sensitivity/specificity value of 80/85%, 80/78%, 80/71%, and 70/78%, respectively. (**B**) The AUC for the miRNA panel was 0.82 (95% CI (0.50-0.94), cut-offs 0.82, with a sensitivity of 85.7%, and a specificity of 80% (logit model formula: –2.34 + 2.48 * miR-92a-3p + 1.47 × miR-151a-5p + 1.97 × miR-106b-5p - 2.55 × miR-93-5p. OBS+, obese subjects with steatosis; PWS+, Prader–Willi Syndrome with steatosis AUC, area under the ROC curve; CI, confidence interval.

The diagnostic potential of each significant miRNAs in discriminating PWS+ vs. OBS+ grade 1 was further evaluated. Independent ROC curve analysis showed a solid individual performance of each miR-151a, mir-92a-3p, miR-106b-5p, and miR-93-5p with AUC values of 0.85 (0.56–0.95, 95% CI), 0.79 (0.50–0.92, 95% CI), 0.75 (0.46–0.89, 95% CI), and 0.77 (0.49–0.90, 95% CI), respectively (Figure 2A). At the established diagnostic cut-off values, the optimal sensitivity/specificity for miR-151a, miR-92a-3p, miR-106b-5p, and miR-93-5p were 80/85%, 80/78%, 80/71%, and 70/78%, respectively. One-way stepwise logistic regression analysis of the combination between the four significant miRNAs showed an AUC value of 0.82 (95% CI (0.50–0.94)) at the established cut-offs of 0.82, with a sensitivity of 85.7%, and a specificity of 80% (logit model formula: –2.34 + 2.48 × miR-92a-3p + 1.47 × miR-151a-5p + 1.97 × miR-106b-5p - 2.55 × miR-93-5p) (Figure 2B). Interestingly, the combination of the significant miRNAs never reached superior performance in comparison to miR-151a alone on discriminating PWS+ from OBS+ grade 1. When considering grade 2/3 of steatosis in both populations, no significant differences were observed among the miRNAs analyzed (Supplementary Figure S1).

### 3.5. Differently Expressed miRNAs between Grade1 and Grade 2/3 Steatosis in PWS+ Group

The absence of statistically significant differences between grade 2/3 PWS+ and OBS+ were explained when comparing the differences between the different grades of steatosis within the same patients group. As a general observation, the levels of most of the miRNAs were increased according to the grade of steatosis in PWS+. However, only miR-93-5p and miR-106b-5p reached the statistical significance with the mean fold of change of 2.7 (*p* < 0.05) and 3.48 (*p* < 0.05), respectively (Table 3, Figure S1C,D).

**Figure 3:**
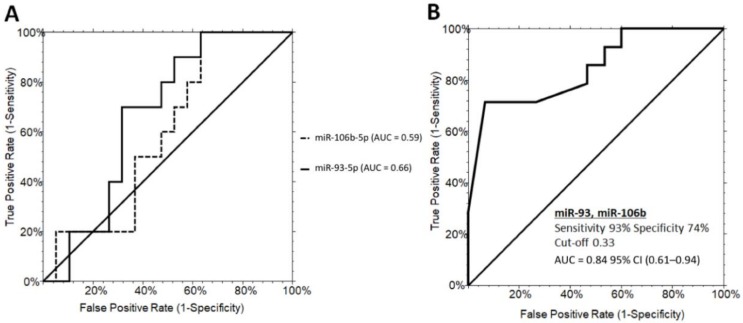
ROC curve analysis of serum miRNAs discriminating PWS+ grade 1 and PWS+ grade 2/3. (**A**) ROC curves for miR-93-5p (60% sensitivity and 52% specificity) and miR-106b-5p (70% sensitivity and 68% specificity). (**B**) ROC curves illustrating the performance of the miRNA panel in discriminating grade 1 from grade 2/3 steatosis in PWS+. PWS+, Prader–Willi Syndrome with steatosis AUC, area under the ROC curve; CI, confidence interval.

Although they were not statistically significant, miR-151a-5p, miR-92a-3p, miR-425-5p, and miR-191-5p were upregulated in grade 2/3 steatosis (Supplementary Figure S1A,B,F,G). In addition, miR-106b-5p, miR-93-5p, miR-122-5p, and miR-425-5p showed an increased intra-variability within the grade 2/3 PWS+ group (Supplementary Figure S1C–F). ROC curves for the miR-93-5p and miR-106b-5p did not reach satisfactory performances with AUC values of 0.59 (0.34–0.76, 95% CI) and AUC values of 0.66 (0.42–0.82, 95% CI), respectively (Figure 3A). However, combining miR-93-5p and miR-106b-5p in a two-way stepwise regression analysis, the AUC value increased to 0.84 (0.61–0.94, 95% CI) at a cut-off determined at 0.33 with a sensitivity of 93% and specificity of 74% (logit model formula: 0.73 + 4.89 × miR-93-5p - 4.61 × miR-106b-5p - 5.90 × miR-93-5p × miR-93-5p + 6.38 × miR-93-5p × miR-106b-5p - 0.88 × miR-106b-5p × miR-106b-5p) (Figure 3B).

### 3.6. Differently Expressed miRNAs in Relation to the Genetic Subtypes of PWS

The expression of serum miRNA panel in relation to the genetic subtypes of PWS (del15 vs. UPD15) was also investigated. No statistically significant differences were detected among the considered miRNAs, with the exception of miR-93-5p that showed a 2.38-fold decrease (*p* < 0.05) in subjects with the chromosome 15 deletion (Supplementary Table S3).

## 4. Discussion

The regulatory function of miRNAs has emerged as an important cellular process in a variety of diseases. MiRNAs are abundantly expressed in the liver, where they exert crucial roles in many metabolic pathways, including lipid metabolism [23]. MiRNAs can be released in the bloodstream by liver cells through both passive and active mechanism. The availability of these small non-coding RNAs in blood have promoted their potential as circulating biomarkers. Despite the presence of many studies focusing on the role of miRNAs as biomarkers, limited information exists in regards of rare diseases, such as PWS. In a previous work by our group, the circulatory miRNome characterizing the PWS in comparison to non-syndromic obesity [17] was explored, giving the hint for following studies aiming to define the clinical significance of circulatory miRNAs in this rare disease. By extending our research to a larger group of subjects, in this study we aimed to identify miRNA biomarker candidates characterizing PWS and OB subjects in relation to the different stages of liver steatosis. By validating the selected miRNA signature distinguishing the two groups of patients, the previously observed differences [17] were confirmed, although only miR-122- 5p, miR-151a-5p, and mir-92a-3p resulted statistically significant. The down-regulation of these miRNAs seem to characterize the PWS subjects. In fact, when included in a step-wise logistic regression model, the three miRNAs reached a sensitivity of 77.7% and a specificity of 71.4 (AUC = 0.81) in distinguishing PWS from OB. Interestingly, all these differently expressed miRNAs are somehow related to the lipid metabolism, storage, and mobilization. As previously discussed [17], miR-122-5p is one of the hepatic hallmarks with key roles in lipid metabolism and trafficking. The molecular function of miR-92a-3p, previously known as mir-92a, was recently elucidated in a work by Niculescu LS et al. [24] in which, by targeting ABCG4 and NPC1, miR-92-3p was shown to impair cholesterol efflux from cells. In particular, ABCG4 is responsible for the cholesterol loading into HDL particles [25] and its inhibition causes a defective cholesterol efflux to HDL [26]. The inhibition of miR-92-3p and the concomitant increased expression of the two targets reduced cholesterol levels in liver and plasma [24]. Interestingly, PWS subjects with lower mir-92a-3p expression show significantly higher HDL cholesterol levels (*p* = 0.01), although no significant correlation was found.

Mir-92a-3p, which is reported to be inversely correlated with the human brown adipose tissue (BAT) activity [27], has been suggested as a potential serum biomarker for BAT in mice and humans. A mouse model with a deletion of the imprinting center (PWS-IC^del^ mouse), shows an increased lipid utilization in BAT [28], however this experimental model is characterized by an incomplete phenotype of the syndrome, with reduced body weight. On the other hand, no data about BAT are currently available in patients with PWS.

The expression of serum mir-151a-5p, which has been already associated with dyslipidemia, (defined according to World Health Organization criteria: triglycerides ≥ 1.7 mmol/L and HDL-C <  0.9 mmol/L for men or < 1.0 mmol/L for women) in obese subjects [29], was very low in our PWS subjects. This finding is in line with the better lipid profile of PWS patients in comparison with obese subjects. This difference could involve not only lipid profiles but also lipid accumulation in the liver. Indeed, Golding et al. [28] showed that the hepatic lipid storage in a PWS-IC^del^ mice was disrupted with a fewer but larger lipid droplets compared to the wild type. It is worth noting the finding of major differences in PWS compared to OB when considering the presence of steatosis grade 1. Again, miR-151a-5p and miR-92a-3p were statistically significant, together with the other two circulating miRNAs, miR-106b-5p and miR-93-5p. At lower stages of steatosis, the expression of these four miRNA is reduced in PWS with a lower variability among samples, yet their expression levels were increased in PWS with steatosis grade 2 and 3 at levels comparable with the OB group. These observations are consistent with the performances of these miRNAs in distinguishing OB from PWS subjects (AUC = 0.82, 85.7% sensitivity, 70% specificity). Interestingly, the performances of miR-151a-5p alone are even better with the AUC of 0.85 with a specificity and sensitivity of 80% and 85%, respectively, opening new perspectives for research involving miR-151a-5p in obesity-related diseases. 

The changes in the miRNA profile in PWS according to the degree of steatosis involved other two miRNAs, miR-106b-5p and miR-93-5p, which significantly increased in PWS with steatosis grade 2 and 3. This finding suggests a possible role of these miRNAs for a better classification of the PWS subpopulation, in addition to the already discussed role in adipose tissue and adipogenesis [17]. The combination of the two miRNAs reached an AUC of 0.84 (93% sensitivity and 74% specificity) in distinguishing the patients with a different grade of steatosis. Altogether, these data indicate the presence of some differences between the two groups especially at the initial stage of steatosis. Other groups investigated the correlation between circulating miRNA profiles and progression of steatosis in non-syndromic subjects [30,31]. As expected, the circulating signatures were different from the ones we observed in PWS. In spite of the difference in the miRNA profiles, the studies agree on the correlation of miR-122-5p with the severity of the disease. In our study, the trend was consistent with those findings, but was not statistically significant.

There are some limitations to our study. First, the presence of hepatic steatosis was not confirmed histologically. However, ultrasonography is considered as a reliable method to assess the presence and the different grades of liver steatosis that obviates to liver biopsies [32,33,34]. Without doubt, the histological confirmation of steatosis would have added some values. However, three different reasons hampered us to perform this measurement in our study group. The first one was related to the failed approval by our Ethical Committee, the second one was the additional costs for the huge number of subjects recruited for the present study that were already burden with elevated costs and last, but not least, the weak compliance by patients suffering with Prader–Willi syndrome to be diagnosed with invasive methods.

A second weakness is that the sample size of our PWS group is relatively small. However, it should be remembered that PWS is a rare disease and enrolment of these patients is difficult. Another problem is related to the lack of data on follow-up of our patients, as our study is exclusively cross-sectional. In this context, more research is needed to assess the clinical course of liver steatosis during adulthood in PWS, particularly in older ages. Even so, this study provides new knowledge to describe the clinical picture of PWS and to increase awareness of the syndrome. In addition, these data could be useful to better characterize the development of liver steatosis both in subjects with syndromic obesity and in the general population.

## Figures and Tables

**Table 1:** Relative mean ΔΔCq expression of serum miRNAs of obesity (OB) and Prader–Willi syndrome (PWS) subjects.

MiRNA	OB Subjects Mean (Range)	PWS Subjects Mean (Range)	Fold Change
** hsa-miR-122-5p	0.70 (0.22–1.17)	0.16 (0.09–0.22)	4.38
* hsa-miR-151a-5p	0.90 (0.52–1.28)	0.33 (0.16–0.51)	2.72
* hsa-miR-92a-3p	0.71 (0.52–0.89)	0.53 (0.28–0.78)	1.34
hsa-miR-425-5p	1.39 (0.89–1.89)	0.76 (0.46–1.07)	1.82
hsa-miR-23a-3p	1.36 (0.83–1.89)	1.02 (0.64–1.40)	1.33
hsa-miR-24-3p	1.03 (0.59–1.48)	0.78 (0.36–1.20)	1.32
hsa-miR-93-5p	0.68 (0.38–0.98)	0.56 (0.23–0.89)	1.21
hsa-miR-106b-5p	0.85 (0.54–1.16)	0.76 (0.36–1.16)	1.11
hsa-miR-191-5p	0.77 (0.51–1.02)	0.69 (0.34–1.04)	1.11

**p < 0.01 * *p* < 0.05, fold of change is reported as OB mean exp/PWS mean exp.

**Table 2:** Relative mean ΔΔCq expression of serum miRNAs of grade 1 PWS+ subjects and OBS+ subjects.

MiRNA	OBS+ Gr. 1 Mean (Range)	PWS+ Gr. 1 Mean (Range)	Fold Change
*** hsa-miR-151a-5p	1.11 (0.47–1.74)	0.38 (0.05–0.71)	2.92
** hsa-miR-92a-3p	0.88 (0.59–1.17)	0.46 (0.13–0.78)	1.91
* hsa-miR-106b-5p	0.92 (0.37–1.47)	0.52 (0.01–1.08)	1.76
* hsa-miR-93-5p	0.78 (0.38–1.19)	0.49 (0.01–1.10)	1.59
hsa-miR-122-5p	0.25 (0.06–0.45)	0.14 (0.03–0.24)	1.78
hsa-miR-425-5p	1.25 (0.38–2.12)	0.74 (0.26–1.23)	1.68
hsa-miR-24-3p	1.10 (0.42–1.78)	0.68 (0.01–1.36)	1.61
hsa-miR-191-5p	0.85 (0.40–1.31)	0.67 (0.04–1.30)	1.26
hsa-miR-23a-3p	1.37 (0.64–2.11)	1.17 (0.52–1.83)	1.17

***p < 0.005, ** *p* < 0.01, * *p* < 0.05, fold of change is reported as OBS+ mean exp/PWS+ mean exp.

**Table 3:** Relative mean ΔΔCq expression of serum miRNAs in PWS+ Grade 1 and PWS+ grade 2/3.

MiRNA	Prader–Willi Syndrome		
	Steatosis Gr. 1 Mean (Range)	Steatosis Gr. 2/3 Mean (Range)	Fold Change
* hsa-miR-106b-5p	0.29 (0.01–0.60)	1.01 (0.40–1.61)	3.48
* hsa-miR-93-5p	0.23 (0.21–0.44)	0.62 (0.26–0.98)	2.69
hsa-miR-92a-3p	0.34 (0.11–0.57)	0.88 (0.19–1.58)	2.58
hsa-miR-425-5p	0.59 (0.21–0.97)	1.48 (0.28–2.68)	2.50
hsa-miR-191-5p	0.39 (0.15–0.64)	0.96 (0.31–1.60)	2.46
hsa-miR-151a-5p	0.52 (0.01–1.08)	0.92 (0.37–1.47)	1.73
hsa-miR-23a-3p	1.06 (0.40–1.72)	1.38 (0.20–2.56)	1.30
hsa-miR-122-5p	0.14 (0.03–0.24)	0.17 (0.07–0.27)	1.21
hsa-miR-24-3p	0.38 (0.01–1.36)	0.44 (0.08–0.81)	1.15

*p < 0.05, fold of change is reported as Steatosis Gr.2–3/Gr.1

## Supplementary Materials

The following are available online at https://www.mdpi.com/2077-0383/9/4/1123/s1. Figure S1: Circulating miRNA expression levels in PWS+ and OBS+ subjects according to degree of steatosis. Serum levels of miR-mir-151a (**A**), mir-92a-3p (**B**), miR-106b-5p (**C**), and miR-93-5p (**D**) were upregulated in OBS+ grade 1 in comparison to PWS+ grade 1. PWS+ miR-106b-5p and miR-93-5p were significantly upregulated in PWS+ grade 2/3. There were no significant differences in the expression of miR-122-5p (**E**), miR-425-5p (**F**), miR-191-5p (**G**), miR-23a-3p (**H**), and miR-24-3p (**I**) in both groups. Data are shown as a mean fold of change expression and were normalized to miR-1275. Statistically significant differences were determined by Kruskal–Wallis test using one-way ANOVA procedure. OBS+, non-syndromic obese subjects with steatosis; PWS+, Prader–Willi syndrome with steatosis; ****p* < 0.005, ***p* < 0.01, **p* < 0.05. Table S1: Clinical characteristics of PWS and OB subjects. Table S2: Blood parameters of PWS and OB subjects. Table S3: Relative serum miRNAs expression according to genetic subtypes of PWS patients.
