# Supplementary material for: Circulating microRNA Associated to Different Stages of Liver Steatosis in Prader–Willi Syndrome and Non-Syndromic Obesity
Source: J Clin Med. 2020 Apr 14;9(4):1123. doi: 10.3390/jcm9041123 (PMC7230920; doi:10.3390/jcm9041123)
Supplement: Supplementary file 1 [file jcm-09-01123-s001.zip › Sup tables Pascut.docx]

**Table 1.** Clinical characteristics of PWS and OB subjects.

|  | **OB Subjects** | **PWS Subjects** | ***p* Values** |
| --- | --- | --- | --- |
| ***n*.** | 30 | 30 | / |
| **Degree of liver steatosis 1/2/3** | 10 / 8 / 12  33% / 27% / 40% | 15 / 11 / 4  50% / 37% / 13% | / |
| **Karyotype (del15/UPD/met+)** | / | 21 / 7 / 2 | / |
| **Sex (m/f)** | 15 / 15 | 10 / 20 |  |
| **Age (years)*** | 37.7 ± 7.9 | 35.0 ± 9.9 | 0.25 |
| **Height (m)*** | 1.68 ± 0.1 | 1.50 ± 0.1 | < 0.001 |
| **Weight (kg)*** | 112.1 ± 23.7 | 89.8 ± 26.3 | 0.001 |
| **BMI (kg/m^2^)*** | 41.0 ± 3.3 | 39.8 ± 10.7 | 0.57 |
| **Fat mass (%)*** | 45.5 ± 5.2 | 48.2 ± 7.7 | 0.11 |
| **Fat free mass (%)*** | 54.2 ± 5.8 | 50.9 ± 7.2 | 0.06 |
| **Waist circ. (cm)*** | 118.8 ± 12.9 | 118.2 ± 18.6 | 0.87 |
| **Hip circ. (cm)*** | 124.9 ± 8.0 | 120.2 ± 20.0 | 0.23 |
| **Systolic blood pressure (mmHg)*** | 126.8 ± 14.7 | 126.5 ± 10.1 | 0.92 |
| **Diastolic blood pressure (mmHg)*** | 80.8 ± 7.0 | 79.3 ± 7.4 | 0.42 |

*(expressed as mean ± standard deviation)

PWS, Prader-Willi Syndrome; OB, non-syndromic obese; del15, deletion in the chromosome 15; UDP, Uniparental Disomy; met+, positive methylation test.

**Table 2.** Blood parameters of PWS and OB subjects.

|  | **OB Subjects** | **PWS Subjects** | ***p* Values** |
| --- | --- | --- | --- |
| **AST (U/L)** | 23.3 ± 9.2 | 17.8 ± 5.2 | 0.01 |
| **ALT (U/L)** | 30.6 ± 17.3 | 20.9 ± 10.7 | 0.01 |
| **GGT (U/L)** | 40.6 ± 42.5 | 21.6 ± 11.8 | 0.02 |
| **Glycaemia (mg/dL)** | 92.6 ± 15.4 | 106.6 ± 40.0 | 0.08 |
| **Insulin (mU/L)** | 19.6 ± 9.4 | 11.9 ± 7.7 | < 0.001 |
| **HbA1c (%)** | 5.6 ± 0.5 | 6.2 ± 1.5 | 0.04 |
| **Total cholesterol (mg/dL)** | 184.8 ± 32.3 | 183.5 ± 30.1 | 0.87 |
| **HDL cholesterol (mg/dL)** | 44.0 ± 9.4 | 52.8 ± 13.7 | 0.01 |
| **LDL cholesterol (mg/dL)** | 125.1 ± 28.6 | 123.2 ± 28.9 | 0.80 |
| **Triglycerides (mg/dL)** | 142.7 ± 50.2 | 106.1 ± 36.0 | < 0.001 |
| **Uric acid (mg/dL)** | 6.3 ± 1.0 | 5.8 ± 1.5 | 0.14 |
| **Homocysteine (mmol/L)** | 15.6 ± 5.9 | 12.6 ± 4.0 | 0.03 |
| **Vitamin D (ug/L)** | 20.9 ± 10.9 | 33.3 ± 10.6 | < 0.001 |
| **IGF-I (ug/L)** | 146.3 ± 63.9 | 125.0 ± 62.2 | 0.20 |

(All parameters are expressed as mean ± standard deviation)

PWS, Prader-Willi Syndrome; OB, non-syndromic obese.

**Table 3. Relative serum miRNAs expression according to genetic subtypes of PWS patients.**

| MiRNA | PWS | |  |
| --- | --- | --- | --- |
|  | **UPD15** | **DEL15** | **Fold Change** |
| *hsa-miR-93-5p | 0.94 (0.01-1.93) | 0.40 (0.11-0.67) | 2.35 |
| hsa-miR-151a-5p | 0.51 (0.01-1.03) | 0.37 (0.10-0.64) | 1.35 |
| hsa-miR-92a-3p | 0.67 (0.20-1.13) | 0.67 (0.16-1.18) | 0.00 |
| hsa-miR-122-5p | 0.12 (0.04-0.21) | 0.17 (0.07-0.27) | 0.70 |
| hsa-miR-106b-5p | 1.10 (0.24-1.97) | 0.62 (0.15-1.08) | 1.77 |
| hsa-miR-425-5p | 1.06 (0.33-1.78) | 1.13 (0.26-2.00) | 0.93 |
| hsa-miR-23a-3p | 1.35 (0.35-2.36) | 1.25 (0.37-2.13) | 1.08 |
| hsa-miR-191-5p | 1.07 (0.11-2.03) | 0.70 (0.11-2.03) | 1.52 |
| hsa-miR-24-3p | 1.10 (0.17-2.02) | 0.63 (0.14-1.11) | 1.74 |

**p value<0.01 *p value<0.05.

PWS, Prader-Willi Syndrome; OB, non-syndromic obese; del15, deletion in the chromosome 15; UDP, Uniparental Disomy
