# Supplementary figures and images for: Circulating microRNA Associated to Different Stages of Liver Steatosis in Prader–Willi Syndrome and Non-Syndromic Obesity
Source: J Clin Med. 2020 Apr 14;9(4):1123. doi: 10.3390/jcm9041123 (PMC7230920; doi:10.3390/jcm9041123)

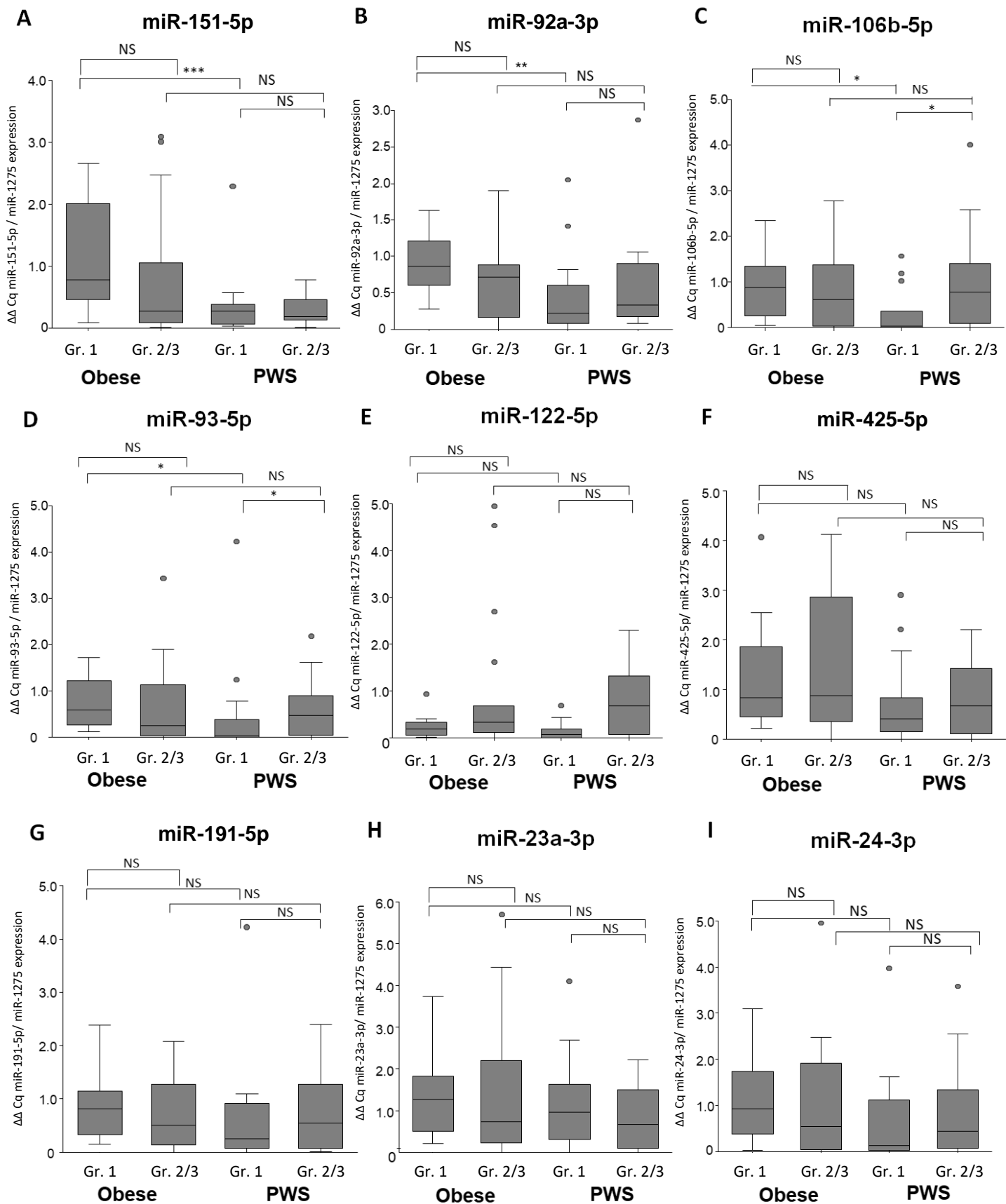

Supplement: Supplementary file 1 [file jcm-09-01123-s001.zip › SUPPL FIG 1 Pascut.pdf]
